# Poland Syndrome: Use of Vertical Expandable Prosthetic Titanium Rib System before Walking Age—A Case Report

**DOI:** 10.1055/s-0036-1593354

**Published:** 2016-09-14

**Authors:** Rosen S. Drebov, Atanas Katsarov

**Affiliations:** 1 Department of Pediatric Surgery, University Emergency Hospital “N.I. Pirogov”, Sofia, Bulgaria; 2 Department of Children's Orthopaedics, University Emergency Hospital “N.I. Pirogov”, Sofia, Bulgaria

**Keywords:** Poland syndrome, VEPTR, vertical expandable prosthetic titanium rib

## Abstract

**Aim**  To present a new therapy for Poland syndrome (PS) using a novel surgical approach: the vertical expandable prosthetic titanium rib (VEPTR) system.

**Methods**  The VEPTR system rib-to-rib variant was used to enhance the chest wall and vertebral column support in a young patient before walking age.

**Case Report**  We present a 12-month-old infant diagnosed with left-sided PS at the age of 6 months associated with missing ribs, scoliosis, and absence of the left pectoral muscles. Because of four missing ribs, paradoxical breathing was present. In addition, the left scapula was protruding into the chest due to the missing rib support. Scoliosis was caused by a left-sided nonsegmented bar of the thoracic spine.

**Results**  We decided to use the VEPTR system before the patient reached walking age to prevent progression of column deformation and future pulmonary problems. To improve the spinal deformity, to stabilize the thorax, and to improve thoracic function, we performed the operation at 1 year of age. At 10-month follow-up, the patient was reevaluated. The construction was still stable and scoliosis had not deteriorated.

**Conclusion**  The VEPTR system is a choice of treatment in young patients with PS to prevent late complications after a child reaches walking age.

 Poland syndrome (PS), also known as Poland sequence or Poland anomaly, displays many distinctive features. 1 It was initially reported by Sir Alfred Poland in 1841. 2 PS presents with various thoracic deformities. 

## Aim

To present a novel approach in the treatment of PS, using a vertical expandable prosthetic titanium rib (VEPTR) rib-to-rib variant.

## Case Report

**Fig. 1:**
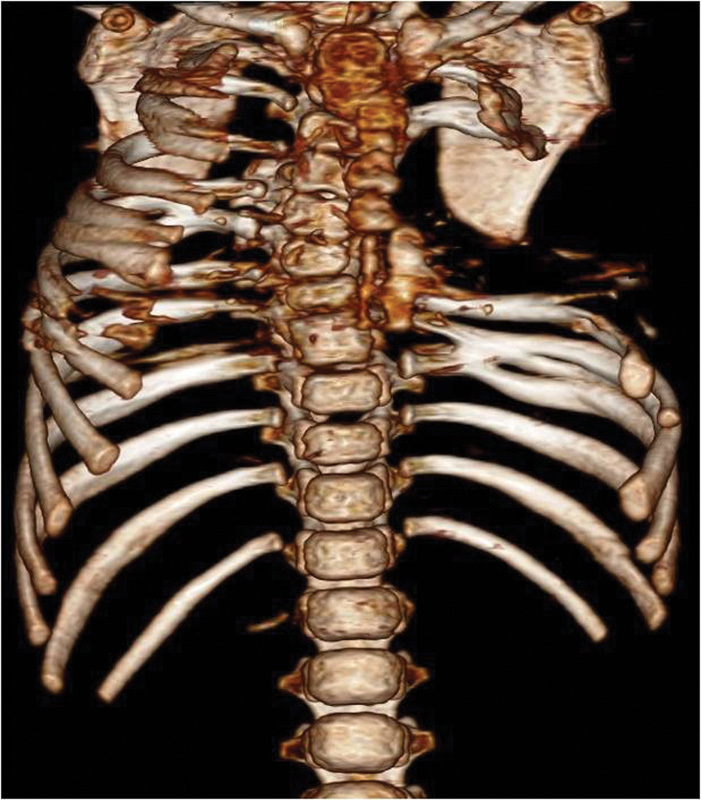
3D computed tomography (CT) reconstruction image of the chest (anterior view).

**Fig. 2:**
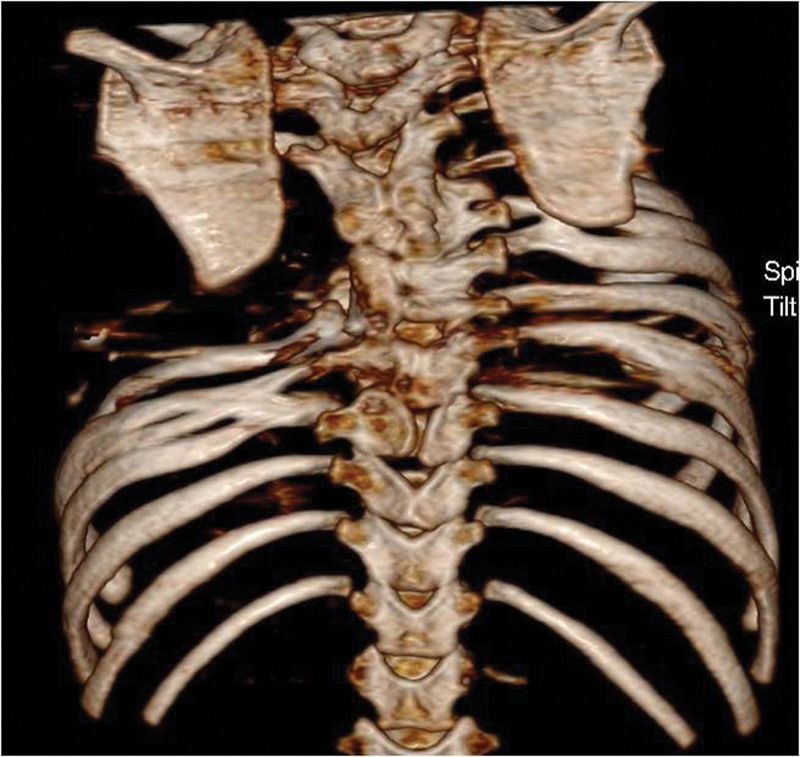
3D CT reconstruction image of the chest and thoracic spine (posterior view).

 We present a 12-month-old infant diagnosed with left-sided PS, diagnosed at the age of 6 months. The clinical presentation included absence of four ribs ( Fig. 1 ), presence of rib fusion, deformities of the thoracic vertebra ( Fig. 2 ), absence of left pectoral muscles, and scoliosis. 

**Fig. 3:**
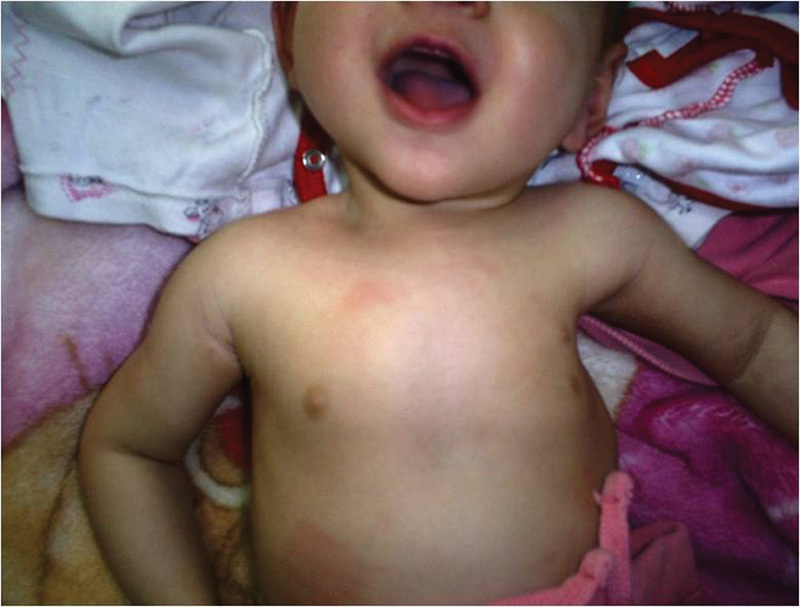
Clinical appearance of the chest malformation and additional breast gland.

 The operation was performed before the patient reached walking age due to the lack of rib support, paradoxical breathing, and the danger of thoracic insufficiency syndrome. The left scapula was protruding into the chest and an aplastic left breast gland was seen. This case was also interesting because of the presence of an additional breast gland ( Fig. 3 ). 

## Methods

**Fig. 4:**
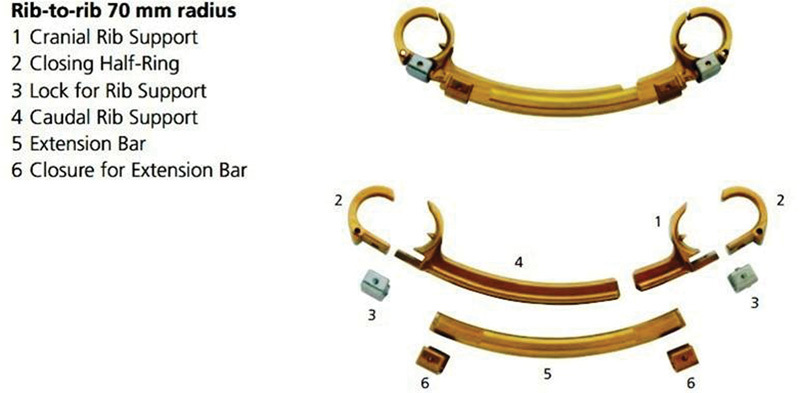
VEPTR device construction.

**Fig. 5:**
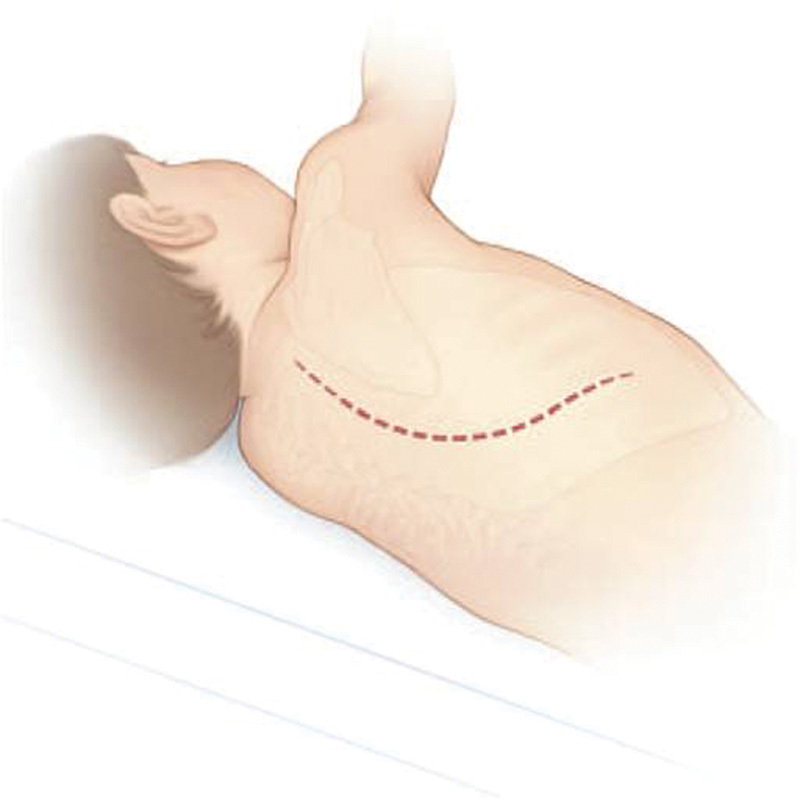
Surgical approach—skin line.

**Fig. 6:**
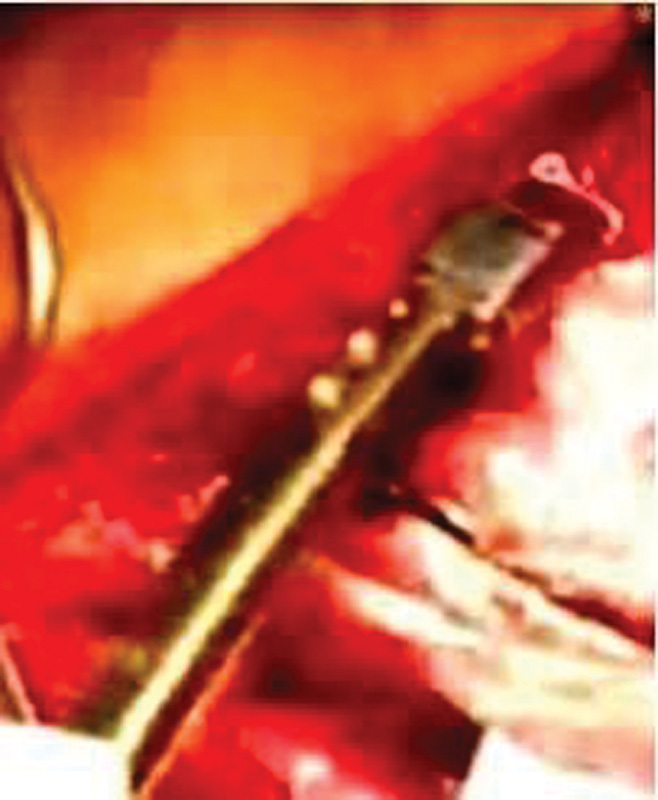
VEPTR device in place.

 We decided to use the VEPTR rib-to-rib system with 70-mm radius before the patient reached walking age to prevent progression of vertebral column deformation and future pulmonary problems, such as thoracic insufficiency syndrome. We performed the first step of the operation when the patient was 1 year old. The system consisted of cranial rib support, closing half-ring, lock for rib support, caudal rib support, extension bar, and closure for the extension bar ( Fig. 4 ). Steps of the first operation included a surgical approach ( Fig. 5 ), mobilization of the sixth and seventh ribs to protect the thoracic area around the missing ribs, placement of the upper and lower rings and lock, insertion of the caudal rib support ( Fig. 6 ), insertion and fixation of extension bar and inside underlying biodegradable patch, and soft tissue repair. 

The second stage of the operation (lengthening of the construction after 1 year) included mobilization of soft tissues around the closure of the extension bar, removal of fixation, distraction of the construction, new fixation, and soft tissue repair.

## Results

**Fig. 7:**
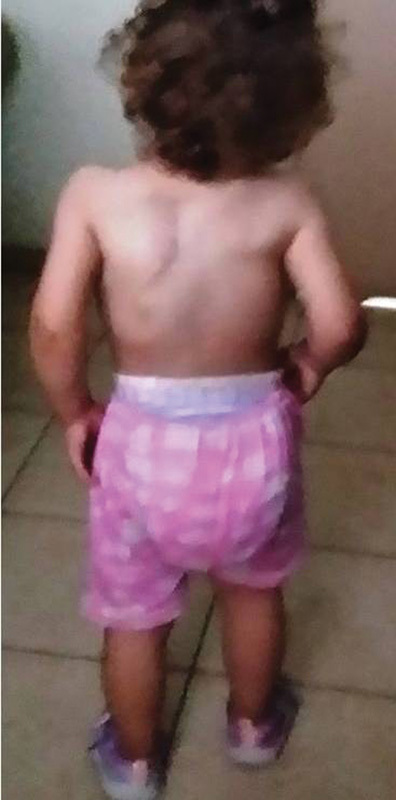
Standing child 10 months after the operation.

**Fig. 8:**
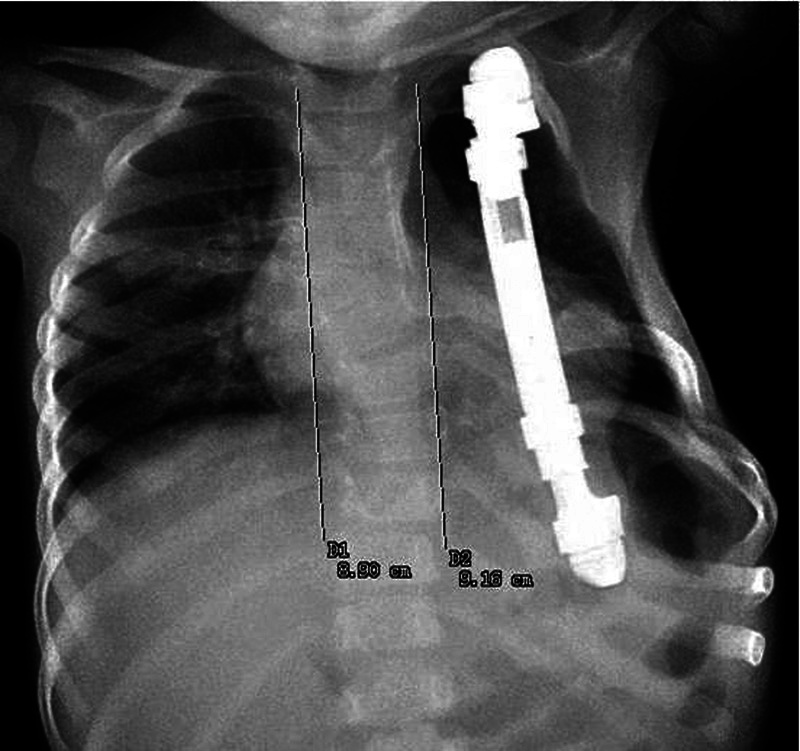
X-ray of the chest 10 months after the operation (anteroposterior view).

**Fig. 9:**
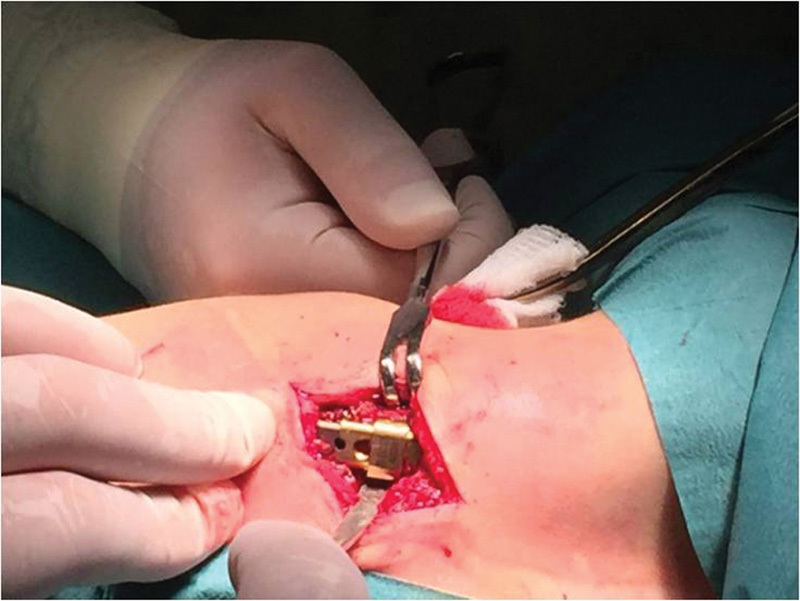
Lengthening the VEPTR device; second operation (1 year after the first).

**Fig. 10:**
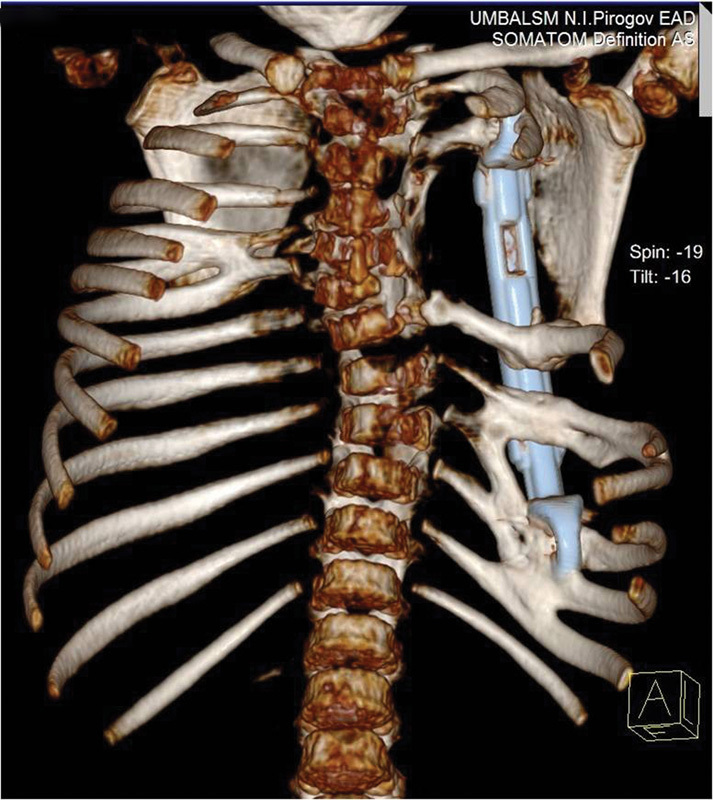
3D CT reconstruction image of the chest with VEPTR device in place, 1 year after the first operation (anterior view).

 Ten months after the first operation, the patient was reevaluated. The construction was still stable, and the scoliosis had not deteriorated ( Figs. 7 , 8 ). After 1 year, growth measurements indicated lengthening the construction ( Fig. 9 ). The result was excellent ( Fig. 10 ). 

## Discussion

 PS is a rare congenital anomaly characterized by unilateral absence of the pectoral muscles and ipsilateral brachysyndactyly, and occasionally it is associated with other malformations of the anterior chest wall and lack of breast and nipple. Very few cases are familial. 3 The most common etiology is suspected to be vascular. 4 The absence of chest wall support leads to scoliosis and thoracic insufficiency syndrome. 

**Fig. 11:**
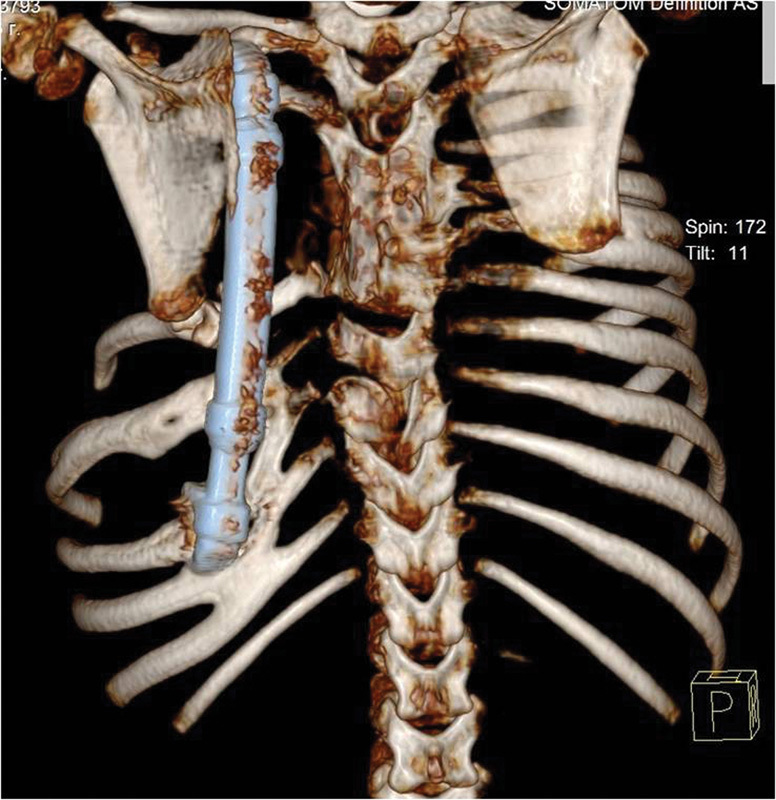
3D CT reconstruction image of the chest with VEPTR device in place, 1 year after the first operation (posterior view). Note the fused ribs.

 Various surgical techniques have been described for repair of chest wall defects in PS. 5 Some authors suggest that the use of autologous material and implants is sufficient for chest wall reconstruction and gives good long-term results. 3 Reconstructive surgery is the main addition to the treatment and includes latissimus dorsi muscle flap and silicone breast implants, 6 but it does not solve the problem of vertebral column support. The VEPTR allows new surgical procedures for treatment of spine deformity in early childhood. VEPTR is not a new “growing rod”; instead, it stabilizes volume-enhancing thoracic reconstructions. 7 8 Lieber et al used the VEPTR system in a complex, single-stage surgery. 5 The main questions from our case involve the behavior of the fused vertebrae in the future ( Fig. 11 ); the chest seems to be stable and if the spine is straight and the VEPTR fits well to the ribs, nothing will need to be done concerning the spine, so it will remain straight. We will continue to observe it closely, with imaging follow-up every 6 to 8 months, and if the spine does not grow straight and starts developing scoliosis again, another VEPTR device will be implanted. 

## Conclusion

The VEPTR system is a novel option for the treatment in very young patients with PS to prevent late complications after the child reaches walking age. According to the literature, our patient is the fifth treated case in the world of PS with this device.
